# Epiphyseal Primary Diffuse Large B-Cell Lymphoma of Bone

**DOI:** 10.1155/2018/4160925

**Published:** 2018-11-26

**Authors:** Shachar Kenan, Leonard Kahn, Morris Edelman, Arlene Redner, Samuel Kenan

**Affiliations:** ^1^Orthopaedic Oncology Fellow, Department of Orthopaedics, Memorial Sloan Kettering Cancer Center (MSKCC), New York, NY, USA; ^2^Musculoskeletal Pathology, Department of Pathology, Zucker/Hofstra North Shore Long Island Jewish, Northwell Health Medical Center, New Hyde Park, NY, USA; ^3^Department of Pediatric Hematology/Oncology, Zucker/Hofstra North Shore Long Island Jewish, Northwell Health Medical Center, New Hyde Park, NY, USA; ^4^Professor, Department of Orthopaedics, Zucker/Hofstra North Shore Long Island Jewish, Northwell Health Medical Center, New Hyde Park, NY, USA

## Abstract

Primary lymphoma of bone (PLB) confined to the epiphysis has only been described in four other patients. Due to the rarity of this entity, diagnosis has often been delayed, leading to mismanagement with adverse clinical consequences. We report a fifth case of primary epiphyseal lymphoma of bone located in the left distal medial femoral epiphysis of a 13-year-old boy. Radiographic and histologic features of PLB are discussed, along with a review of the literature and pitfalls of misdiagnosis. The patient initially presented with six months of progressive left knee pain with an associated loss of passive range of motion. Imaging revealed a mixed radiolucent lesion within the left distal medial femoral epiphysis with cortical breakthrough. A core biopsy was performed revealing a blue round cell tumor. Thanks to modern immunohistochemistry techniques, a diagnosis of primary lymphoma of bone was quickly made. The patient thus avoided further surgical intervention and received the appropriate treatment of chemotherapy, with subsequent rapid resolution of the lesion. This case highlights the necessity of including primary lymphoma of bone in all epiphyseal lesion differential diagnoses, especially in the pediatric patient population when aggressive radiographic features are present.

## 1. Introduction

Lymphoma, when confined to bone at initial presentation, is referred to as primary lymphoma of the bone (PLB). PLB is most commonly diagnosed in patients over 30 years of age with a slight male predominance [1–3]. Patients may clinically present with localized symptoms such as bone pain, swelling, joint contracture, a palpable mass, or fracture. Systemic symptoms may include fever, night sweats, unintentional weight loss, and fatigue, although these symptoms may not always be present, such as in the current case. The predominant subtype is diffuse large B-cell lymphoma (DLBCL); however follicular (FL), small lymphocytic (SLL), marginal zone (MZL), and anaplastic large cell lymphoma (ALCL) have been described [1, 4].

Solitary epiphyseal lesions of bone in children are relatively rare and not easily diagnosed. The radiologic differential diagnosis for this age group includes a broad spectrum of tumor and tumor-like conditions [5]. The most common lesions are chondroblastoma, epiphyseal abscess, and Langerhans cell histiocytosis (LCH). These lesions are generally geographic and do not appear aggressive. Exceptionally rare lesions such as Ewing's sarcoma, osteosarcoma, and epiphyseal PLB may be considered when aggressive radiographic features such as diffuse permeation, periosteal elevation, and cortical breakthrough are seen. In all cases, the clinical and radiologic features should be correlated.

This case report documents an epiphyseal primary lymphoma of bone, diffuse large B-cell subtype, in the left distal femur of a 13-year-old boy. This is the 5^th^ documented case of epiphyseal PLB, a rare diagnosis which has only been described in four other patients to date [6–8].

The authors have obtained parental informed written consent for print and electronic publication of this case report.

## 2. Case Presentation

### 2.1. Clinical

A 13-year-old boy with no past medical history presented with six months of progressive left knee pain. He denied any trauma or constitutional symptoms. On physical exam, the patient had a moderate left knee effusion with some warmth and no erythema. There was tenderness to palpation along the medial joint line and a marked decreased passive range of motion, 40-90 degrees. His neurovascular exam was intact with no signs of lymphedema, adenopathy, or instability.

### 2.2. Imaging

**Figure 1:**
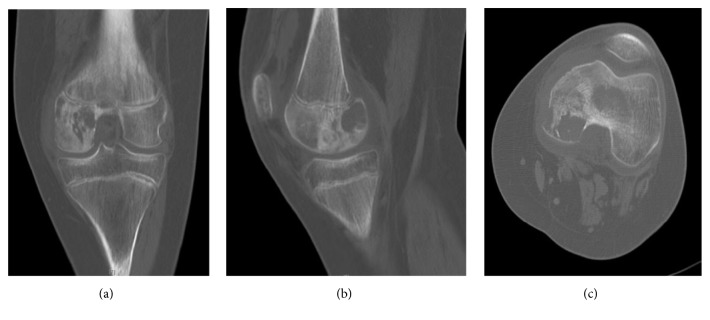
Computed tomography (CT): coronal (a), sagittal (b), and axial views (c) of the left knee, July 2009.

Computed tomography (CT) revealed a mixed radiolucent and sclerotic permeative lesion within the left posterior medial epiphysis with medial cortical destruction (Figure 1). There was an associated suprapatellar effusion without evidence of infiltration through the physis.

**Figure 2:**
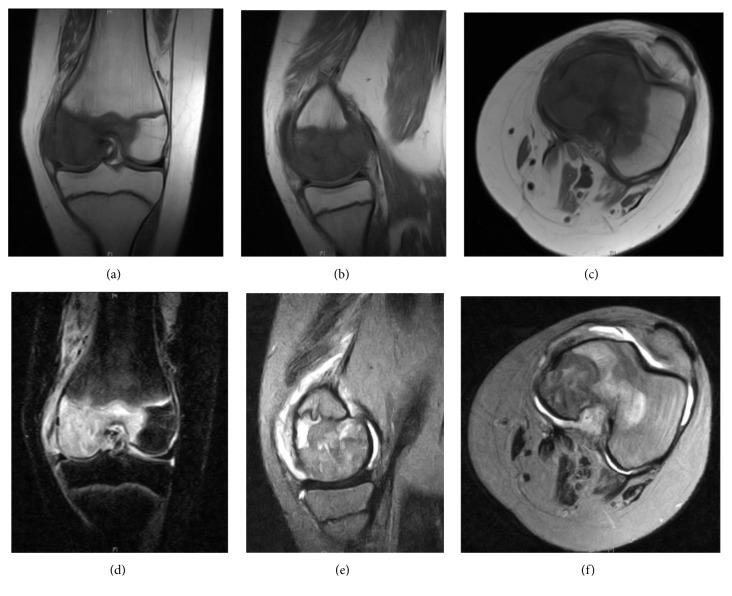
Magnetic resonance images (MRI): T1 coronal, sagittal, and axial views (a, b, c), STIR coronal (d), T2 sagittal (e), and axial views (f) of the left knee, July 2009.

Magnetic resonance imagining (MRI) demonstrated a nonspecific infiltrative process involving the left distal medial femoral epiphysis, extending proximally along the posterior femoral cortical surface, appearing dark on T1 and bright on STIR and T2 weighted images (Figure 2). The radiographic differential diagnosis included chondroblastoma, osteomyelitis and eosinophilic granuloma.

**Figure 3:**
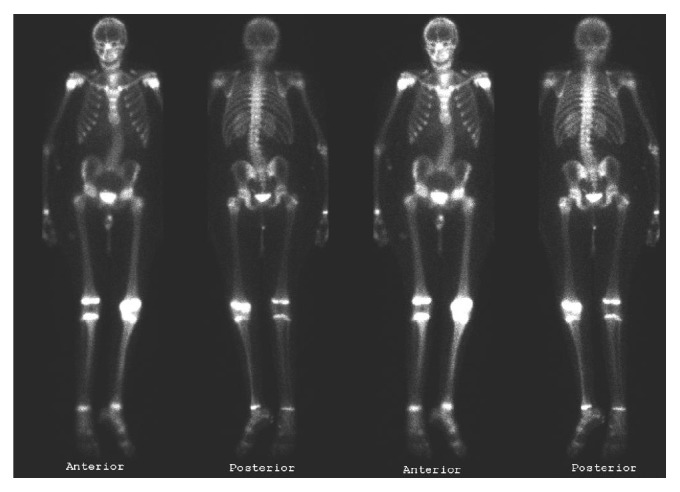
Three-phase bone scintigraphy, August 2009. Increased uptake in the distal left femur on delayed images.

**Figure 4:**
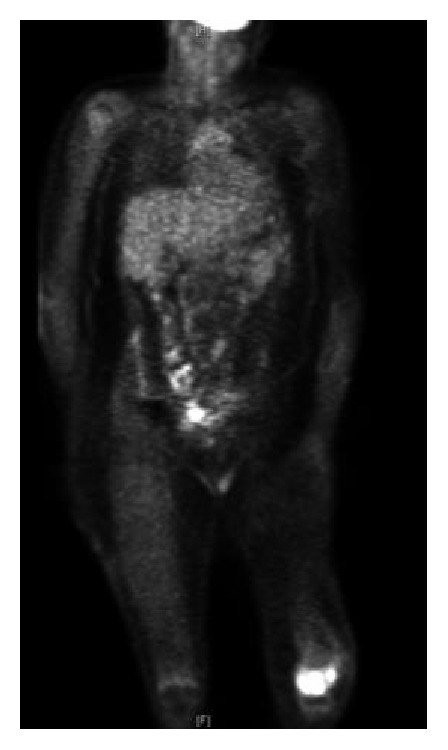
Fludeoxyglucose-positron emission tomography (FDG-PET), August 2009. Increased activity of FDG within the left knee (SUV max of 17.11), with physiologic distribution of FDG in the remainder of the body.

Metastatic workup including CT chest, abdomen, and pelvis, three phase bone scintigraphy, and fludeoxyglucose-positron emission tomography scans (FDG-PET) were all otherwise unremarkable (Figures 3 and 4).

### 2.3. Pathology

**Figure 5:**
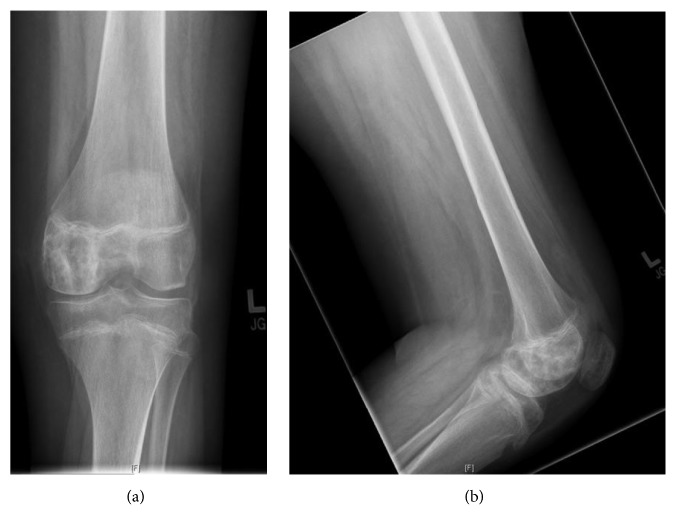
Anteroposterior (a) and lateral radiographs (b) of the left knee after core biopsy, September 2009.

**Figure 6:**
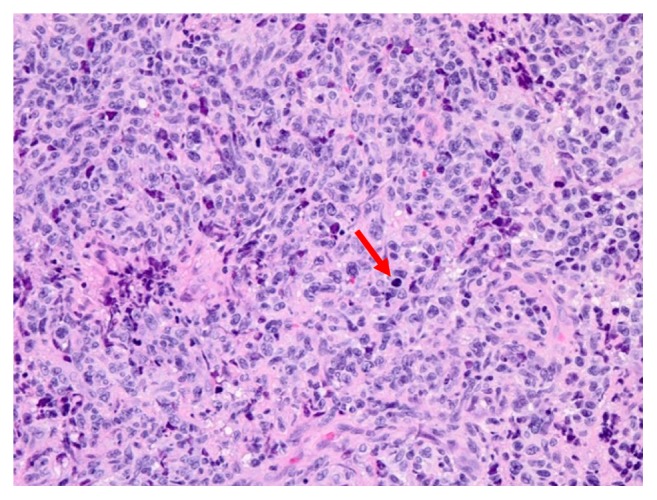
High power magnification, hematoxylin and eosin (H&E) staining. Sheets of dyscohesive atypical cells with large hyperchromatic round nuclei and scant cytoplasm.

**Figure 7:**
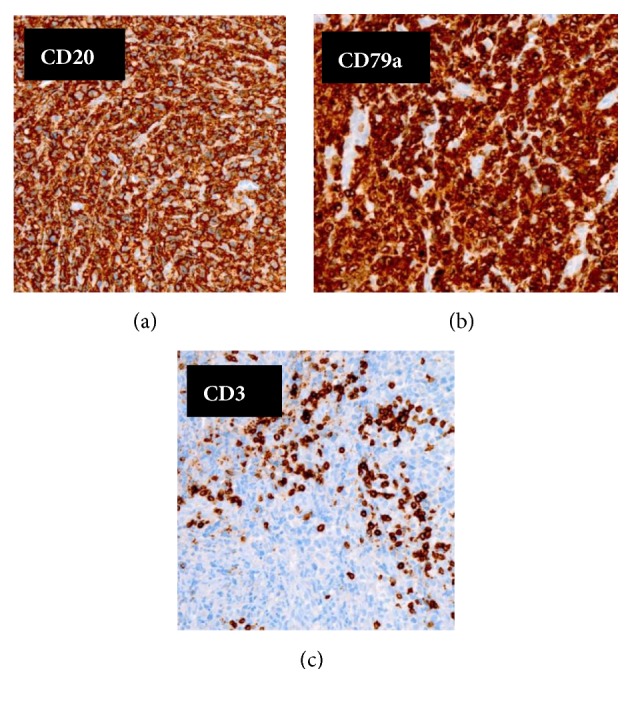
Immunohistochemistry staining. CD20 and CD79a: Strong positive cytoplasmic staining indicative of lymphoid cells of B-type lineage (a, b). CD3: scant scattered reactive T-cells in background (c).

Fluoroscopic guided core biopsy was performed (Figure 5). Histologic analysis revealed a round cell tumor (Figure 6). Immunohistochemistry was positive for CD20 and CD79a, confirming diffuse large B-cell lymphoma (Figure 7). Further analysis revealed positivity for BCL-6 and CD10, and negativity for MUM1, confirming a germinal center phenotype (GC type).

## 3. Discussion

Based on the clinical, radiologic, and histologic features, a diagnosis of epiphyseal primary diffuse large B-cell lymphoma of bone was rendered. The patient subsequently received nine weeks of chemotherapy including vincristine, doxorubicin, cyclophosphamide, and prednisone, without rituximab or radiation therapy (XRT), consistent with prior established regimens for primary lymphoma of bone in children [9].

**Figure 8:**
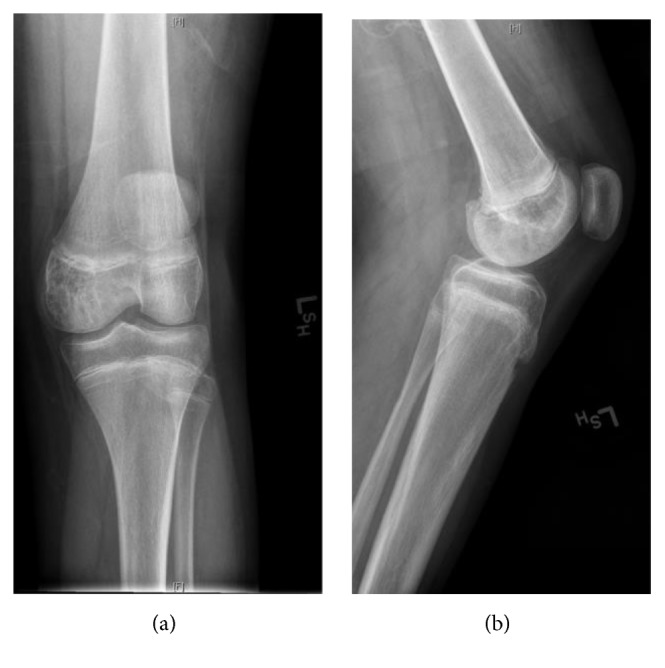
Anteroposterior (a) and lateral (b) radiographs of the left knee after chemotherapy, October 2010. Dramatic lesion response after treatment.

**Figure 9:**
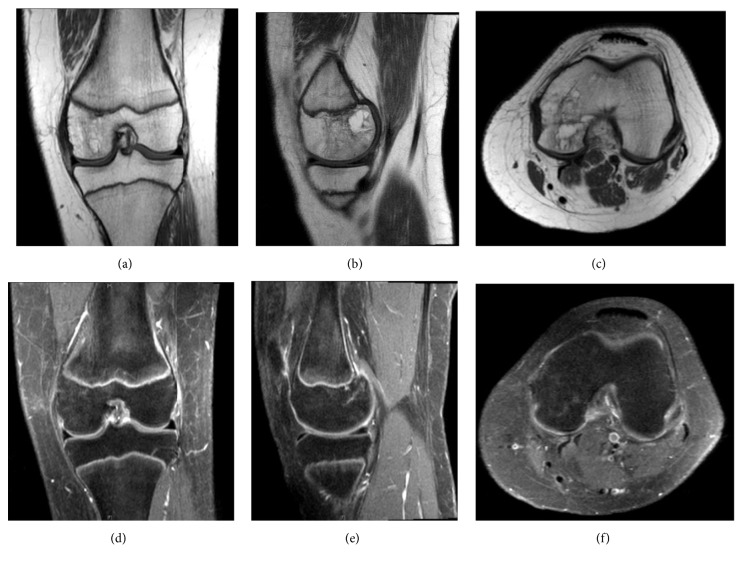
Magnetic resonance images (MRI): T1 coronal, sagittal, axial views (a, b, c) and T1 fat saturated w/contrast coronal, sagittal, axial views (d, e, f) of the left knee after completion of chemotherapy, October 2010. Near complete resolution of lesion.

**Figure 10:**
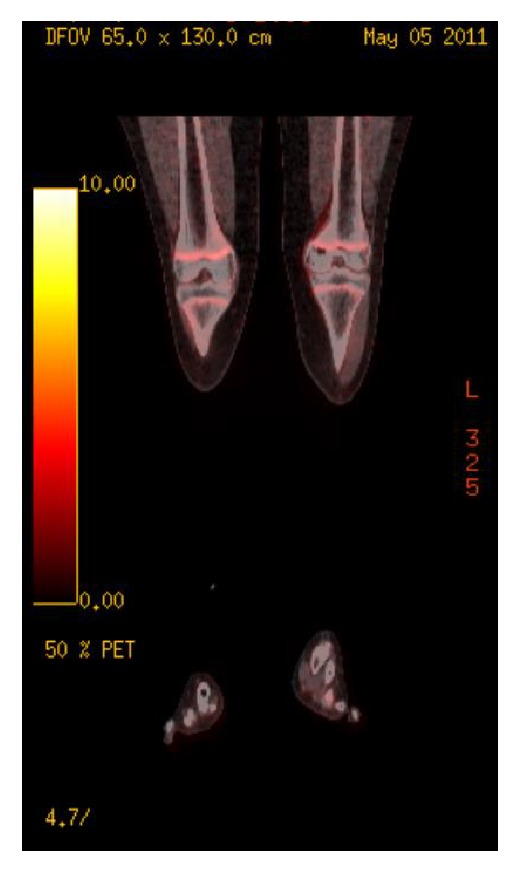
Fludeoxyglucose-positron emission tomography (FDG-PET), May 2011. Non-FDG-avid mixed sclerotic and lytic lesion of the left medial femoral condyle, consistent with resolved lymphoma.

The patient responded well to chemotherapy and his symptoms dramatically improved. At one and a half years after chemotherapy, the patient was in remission and free of pain and had regained full passive knee range of motion. Imaging including MRI and FDG-PET showed a resolving lesion with satisfactory bone remodeling (Figures 8–10).

Primary lymphoma of the bone is rare with an estimated 500 cases reported in the English literature [10–18]. One of the largest retrospective studies described 119 patients with lymphoma involving the musculoskeletal system collected during a period from 1961 to 1999 at Massachusetts General Hospital with a minimum follow-up of 6 months [10]. Patients were divided into four groups based on the World Health Organization classification system [19]: the first group included patients with unifocal osseous disease without lymph node involvement; the second group had multifocal osseous disease without lymph node involvement; the third group had osseous lesions with involvement of multiple visceral sites and the last group included patients who had already been diagnosed with lymphoma prior to osseous involvement [10]. Those patients who met the criteria of the first two groups were given a diagnosis of primary lymphoma of bone, accounting for 70 patients in total, with a median age of 44 years.

Primary lymphoma of bone presents a diagnostic challenge; therefore immunohistochemical techniques play a major role in solving these diagnostic dilemmas. B-cell markers such as CD10, CD20, CD43, CD45, CD79a, CD99, BCL2, BCL6, MUM-1, and terminal deoxynucleotidyl transferase are helpful in establishing a diagnosis [3, 20]. Tissue may be obtained either via image guided percutaneous fine needle, core, or open biopsy. Core biopsy is the method preferred by the authors of this report to ensure an appropriate representative sample is obtained while minimizing damage to surrounding tissue. This is especially important for lymphomas due to the high percentage of tissue necrosis and damage during cytological preparation which reduces diagnostic accuracy [21]. Additionally, care should be taken to avoid iatrogenic injury to open physes during these procedures.

Treatment of primary lymphoma of bone is nonsurgical. Surgery is reserved solely as a palliative measure to restore function and alleviate pain in the rare case of a pathologic fracture [18]. R-CHOP multiagent chemotherapy (rituximab, cyclophosphamide, doxorubicin, vincristine, and prednisone) with or without radiation therapy is the mainstay of treatment in adults [20]. Prognosis is difficult to determine given the rarity of this condition but is generally favorable when treated appropriately, especially in the pediatric population.

A retrospective review from the Moffitt Cancer Center identified 70 patients with confirmed primary lymphoma of bone. These patients had a 3- and 5-year progression-free survival (PFS) rate of 61.2% and 46.9%, respectively, and 5- and 10-year overall survival (OS) rate of 81.1% and 74.7%. Poor prognostic factors included soft tissue extension and multifocality. Researchers at the University of Texas noted a 5-year PFS and OS rate of 80% and 82%, respectively, in their retrospective review of 102 patients with PLB [15]. It is important to note that the majority of the patients in these studies were adults; therefore survival rates are difficult to apply to the pediatric population. The largest pediatric cohort of patients with primary lymphoma of bone included 31 patients collected from 1983 to 1997[9]. At 5-year follow-up, the projected PFS and OS rates were 95% and 100%, respectively. Two-thirds of these children were treated solely with a nine-week course of chemotherapy and only seven received radiotherapy. All 31 of these patients were alive without sequelae; therefore the authors concluded that successful treatment can be obtained without the need for radiotherapy [9].

There have been a total of five reported cases of epiphyseal primary lymphoma of bone including this report. All five involved male patients, aged 8-16 years, presenting within 2-6 months of symptom onset, three cases involved the distal femur, one case involved the proximal tibia, and one case involved the proximal humerus. Four cases were histologically diagnosed as diffuse large B-cell lymphoma and one was diagnosed as anaplastic large cell lymphoma. Misdiagnosis and delay in appropriate treatment may have detrimental consequences, as is highlighted by one of these patients, an 8-year-old boy who presented with a three-month history of right shoulder pain. This patient was subsequently treated with empiric antibiotics for an assumed osteomyelitis after MRI revealed a hyperintense T2 signal within the proximal humeral epiphysis. Eight weeks later, he began complaining of left lower quadrant abdominal pain. A subsequent abdominal CT scan revealed a large mass extending from the left lower abdominal wall into the pelvis. Open biopsy confirmed anaplastic lymphoma of both lesions. A timelier biopsy performed for this patient may have prevented such progression.

## 4. Conclusion

We describe a rare case of epiphyseal primary lymphoma of bone in a 13-year-old boy who presented with six months of left knee pain and an associated flexion contracture. This is the fifth reported case in the medical literature, consistent with the demographics of the previously reported cases. Diagnosis should be made based on the clinical, radiographic and histologic findings with treatment guided by a multidisciplinary team involving orthopaedic and medical oncologists, pathologists, and musculoskeletal radiologists. Biopsy should always be performed when aggressive radiologic features are present. With the appropriate treatment, the prognosis is good. Overtreatment of a medically curable disease such as PLB can lead to significant morbidity and disfigurement. Undertreatment without a timely diagnosis and medical intervention can lead to metastatic disease. Although rare, epiphyseal PLB should be included in the differential diagnosis of any epiphyseal bony lesion, especially in the adolescent population.

## Data Availability

Readers can access the data used in this manuscript by viewing the supporting radiographic images which are included in this study.
